# Analysis of the population structure of *Macrolophus pygmaeus* (Rambur) (Hemiptera: Miridae) in the Palaearctic region using microsatellite markers

**DOI:** 10.1002/ece3.420

**Published:** 2012-11-08

**Authors:** Juan Antonio Sanchez, Michelangelo La Spina, Omaththage P Perera

**Affiliations:** 1 Instituto Murciano de Investigación y Desarrollo Agrario y Alimentario (IMIDA)C/Mayor, 1, La Alberca, 30150, Murcia, Spain; 2 Southern Insect Management Research Unit, USDA-ARSStoneville, MS, 38776, USA

**Keywords:** Evolutionary history, geographic distribution, Mediterranean peninsulas, mirids, molecular diversity, plant bugs

## Abstract

*Macrolophus pygmaeus* (Rambur) (Hemiptera: Miridae) is widely distributed throughout the Palaearctic region. The aim was to explain the current geographic distribution of the species by investigating its genetic population structure. Samples of *M. pygmaeus* were collected in 15 localities through its range of distribution. A sample from a commercial producer was also analyzed. A total of 367 *M. pygmaeus* were genotyped for nine microsatellite loci. Isolation by distance was tested by Mantel's test. The molecular structure of *M. pygmaeus* populations was inferred by UPGMA, AMOVA, Principal component and Bayesian analyses. The average number of alleles per locus per population was 5.5 (range: 3.1–7.8). Istanbul (Turkey) and Nimes (France) had the lowest (0.291) and the highest (0.626) expected heterozygosity (*H*_*e*_), respectively. There was an increase in *H*_*e*_ from the Canary Islands to Nimes, and a progressive decrease thereafter. A significant negative correlation was found between allelic richness and *H*_*e*_, and the distance of each population to the easternmost locality (Canary Islands). Significant linkage disequilibrium was observed in the populations from Turkey. F_ST_ (0.004–0.334) indicated a high population differentiation, with isolation by distance supported by a high correlation. Bayesian analyses, PCA, and UPGMA pointed to three main clusters: (1) Greece and Turkey, (2) Italy and France, and (3) southern Iberia and the Canary Islands. The recent evolutionary history of *M. pygmaeus* is inferred from the data as follows: (1) the reduction in the geographic distribution of the species to the Iberian, Italian, and Balkan peninsulas, and possibly southern France, during glaciations and re-colonization of northern Europe from its southern refuges; (2) the maintenance of high diversity in Iberia and Italy (and possibly southern France) during contraction periods, and bottlenecks in the Balkans; (3) introgression of the Italian–French lineage in northern Spain, naturally or through trade.

## Introduction

**Figure 1:**
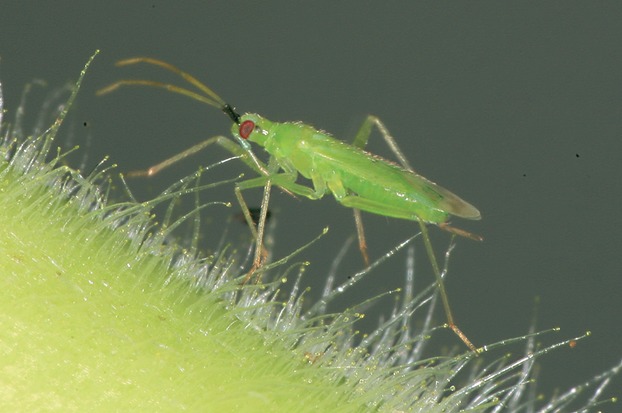
Photograph of a male of *Macrolophus pygmaeus*.

*Macrolophus pygmaeus* (Rambur) (Hemiptera: Miridae) is a zoophytophagous mirid reported on several plant species; many of them belonging to the Solanaceae, Asteracea, and Lamiaceae families (Martinez-Cascales et al. 2006). This plant bug is known to develop and reproduce feeding on some plant species, although its performance is greater feeding on prey than on plants (Lykouressis et al. 2001, 2008; Ingegno et al. 2011). The species reproduce sexually and females insert the eggs into plant tissues leaving only the operculum visible (Gemeno et al. 2007; Perdikis et al. 2003). Nymphs are apterous and go through five developmental instars before becoming alate adults (Fig. 1). Nymphs may walk to neighboring plants and move over short distances, but adults are responsible for the long-range dispersal of the species. *Macrolophus pygmaeus* is a Palaearctic species reported from localities as far East as Tadzhikistan, the Azores Islands in the Atlantic Ocean to the West, Finland to the North, and Algeria to the South (Kerzhner and Josifov 1999). The present natural geographic distribution of the species may indeed be much narrower than that reported based on collection data. There has been much controversy about the taxonomy and species identity of *M. pygmaeus* since the first reports of the species (Martinez-Cascales et al. 2006). The difficulties in taxonomically differentiating sibling species, based on relatively uninformative simple morphological characters of the genus *Macrolophus,* may have lead to misidentification of many of the specimens' labels as “*pygmaeus*” at museum and private collections. The current geographic distribution of *M. pygmaeus* in the Palaearctic region may have been greatly influenced by the alternation of cold and warm climate cycles during the Pliocene, as well as by the geographic barriers interfering with the spreading of the species from its likely southern refuges. The cold periods during the glaciations in the Quaternary are believed to be responsible for much of the current distribution of the European wildlife (Taberlet et al. 1998; Hewitt 1999, 2000). The variation in climate conditions during the glaciations also had a strong impact on vegetation over wide geographic areas (Hewitt 1999; Comes and Kadereit 1998), and thus on the insects associated to that flora (Rousselet et al. 2010; Borer et al. 2012). During cold periods, the three main Mediterranean peninsulas (Iberia, Italy, and the Balkans) were refuges for many species found currently further north in the Palaearctic region (Taberlet et al. 1998; Hewitt 1999; Schmitt and Seitz 2001). The Alps and the Pyrenees were important physical barriers blocking the expansion of animals and plants from their refuges in the Italian and Iberian peninsulas, respectively (Taberlet et al. 1998; Hewitt 1999, 2000). In these Peninsulas, populations of many species diverged genetically due to genetic drift during long periods of isolation during glacial maxima and limited gene flow during interglacial phases (Hewitt 1996; Taberlet et al. 1998; Hewitt 1999; Schmitt 2007).

The likely isolation of *M. pygmaeus* in one or several refuges during the last glaciations, when most of northern and middle Europe were covered either by ice fields or permafrost (Hewitt 2000), might have had an important impact in the past and current population structure of this species. Geographically isolated populations evolved independently and may have become genetically differentiated as the result of local adaptation and genetic drift. This possible divergence in biological and ecological traits during the isolation periods, together with the effect of mountains and seas as physical barriers for dispersion, might have shaped the structure of *M. pygmaeus* populations when the species spread from its southern refuges and colonized new suitable areas after the ice fields were retreated. The trade of *M. pygmaeus* for pest control in protected vegetable crops may also have influenced its current population structure. *Macrolophus pygmaeus* is an economically important beneficial insect released in European greenhouses since 1994 to control small arthropods pests of vegetable crops (Castañé et al. 2011; van Lenteren 2003). The trade of this species has mainly occurred in the European countries (van Lenteren 2003), although the species has been occasionally used for pest control in remote countries, such as New Zealand (Eyles et al. 2008; Flynn et al. 2010) and Malaysia (Mohd-Rasdi et al., 2009). The trade of the species is generally banned in countries where it is not native (e.g., Gillespie et al. 2007). This mirid preys on many arthropod pests such as whiteflies, *Trialeurodes vaporiarorum* (Westwood), and *Bemisia tabaci* (Gennadius) (Hemiptera: Aleyrodidae) (Perdikis and Lykouressis 2000, 2002; Lykouressis et al. 2009; Jaeckel et al. 2011), aphids such as *Myzus persicae* (Sulzer) (Hemiptera: Aphidae) (Perdikis and Lykouressis 2004; Lykouressis et al. 2007; Vandekerkhove et al. 2011), and Lepidoptera, such as *Tuta absoluta* (Meyrick) (Lepidoptera: Gelechiidae) (Urbaneja et al. 2009). In Europe, *M. pygmaeus* has been mainly released in areas with concentration of commercial greenhouses, which represent a small surface of the whole European territory. This plant bug spontaneously colonizes tomato crops surrounded by wild vegetation and with little spray of insecticides, which makes possible to develop biological pest control programs based on the conservation of wild populations of this mirid (Alomar et al. 2002; Sanchez et al. 2003; Martinez-Cascales et al. 2006; Lykouressis et al. 2008). In areas where *M. pygmaeus* is released for pest control, commercial strains might disperse to the neighboring ecosystem and interbreed with wild populations.

*Macrolophus pygmaeus* has been the object of attention of many works from an agricultural and ecological point of view. However, the study of the population structure of the species was never addressed, because no suitable markers were available until the recent development of microsatellite markers by Sanchez, La-Spina, and Perera (Molecular Ecology Resources Primer Development Consortium et al. 2011). Microsatellites have become one of the most used molecular makers for the study of ecological parameters in populations such as migration rates, bottlenecks, population differentiation, ancestry, and relatedness of individuals (Selkoe and Toonen 2006). Microsatellites have been successfully used to determine the genetic variation in populations, speciation, population structure, spatio-temporal genetic variation, and population expansion in *Drosophila melanogaster* (Lachaise) (Nunes et al. 2008), *Thaumetopoea pityocampa* Den. & Schiff. (Santos et al. 2011), *Phlebotomus papatasi* (Scopoli) (Hamarsheh et al. 2009), *Bactrocera oleae* (Gmelin) (Augustinos et al. 2005; Zygouridis et al. 2009), *Cicada* spp. (Hemiptera: Cicadidae)(Seabra et al. 2009), among many other species of insects. Microsatellite markers have been developed for other mirid species (Perera et al. 2007; Shrestha et al. 2007; Kobayashi 2008), but this is the first time that microsatellites are applied to the study of the population structure of a mirid species through an extensive area. In this study, the genetic variation in *M. pygmaeus* populations throughout much of its geographic distribution was explored using a set of nine microsatellites markers. The aim of this study was to investigate the population structure of *M. pygmaeus* and to understand the factors that may have contributed to shaping it.

## Materials and Methods

### Source of *Macrolophus pygmaeus*

A total of 367 *M. pygmaeus* were used in this study, averaging 23 individuals per populations (range: 9–47) (Table 1). Adults were identified primarily based on morphological characters according to Martinez-Cascales et al. (2006) and molecular techniques (amplification and sequencing of mtDNA) were used when morphological characters did not provide satisfactory information for the identification of the species (Martinez-Cascales et al. 2006). Adults were preferably used, but nymphs were taken whenever there were not enough adults in the sample; nymphs were identified by the sequencing of mtDNA according to Martinez-Cascales et al. (2006). A total of 15 populations and one commercial strain were used in this study (Table 1) and Fig. 1 shows the geographic distribution of collection sites. All the collections were made on *Solanum lycopersicum* L. (Solanaceae). The sample from England was collected in a small domestic tomato greenhouse with wide openings, which allow the free movements of insects between the exterior and interior of the greenhouse and where no *M. pygmaeus* releases were effectuated, thus it was considered as a native population.

**Table 1:** Localities and *Macrolophus pygmaeus* sample information

			GPS coordinates			
					
Country	Locality	Code	Latitude	Longitude	Year	*N*
Spain	Tenerife, Canary Islands	Ten-S	28° 23′ 35″N	16° 35′ 41″E	2004	9
Portugal	Castello de Vide	Cas-P	39° 24′ 53″N	7° 27′ 37″E	2006	24
Portugal	Portalegre	Por-P	39° 17′ 31″N	7° 26′ 27″E	2006	24
Spain	Argentona	Arg-S	41° 33′ 15″N	2° 24′ 31″W	2005	20
Spain	Benablon	Ben-S	38° 03′ 27″N	1° 56′ 27″E	2002	47
Spain	Moratalla	Mor-S	38° 12′ 36″N	1° 46′ 12″E	2002	12
Spain	Valentin	Val-S	38° 10′ 58″N	1° 43′ 77″E	2002	33
United Kingdom	Colchester	Col-E	51º 55′ 49″N	0° 59′ 33″W	2003	20
France	Nimes	Nim-F	43° 50′ 09″N	4° 21′ 09″W	2006	20
Italy	Albenga	Alb-I	44° 04′ 39″N	8° 09′ 28″W	2008	26
Italy	Sanda	San-I	44° 22′ 08″N	8° 31′ 42″W	2008	26
Italy	Villarbasse	Vil-I	45° 02′ 42″N	7° 27′ 54″W	2009	23
Greece	Kalampaka	Kal-G	39° 43′ 17″N	21° 38′ 3″W	2007	17
Turkey	Caycuma	Cay-T	41° 20′ 39″N	32° 05′ 19″W	2007	31
Turkey	Istanbul	Ist-T	41° 01′ 22″N	28° 57′ 19″W	2007	15
–	Commercial strain *	C	–	–	2003	20

* This population was originally collected in southern France. *N*= number of individuals genotyped.

### Amplification of microsatellites loci

DNA was extracted using the E.Z.N.A insect DNA Isolation Kit (Omega Bio-tek, Norcross, GA) following manufacturer's instructions. Briefly, *M. pygmaeus* preserved in ethanol were vacuum-dried for 20 min and ground individually in 1.5-mL microcentrifuge tubes in 350 μL of homogenization buffer (Buffer CTL) followed by addition of 25 μL Proteinase K (20 mg/mL) to the homogenates. The samples were mixed briefly and incubated overnight at 37°C. A 24:1 mixture of chloroform: isoamyl alcohol was added to the homogenate, vortexed to mix, and centrifuged at 10,000 ×*g* for 2 min at room temperature. The supernatant of each sample was transferred to a new 1.5-μL microcentrifuge tube and mixed with one volume of Buffer CBL and 2 μL of RNAase solution followed by 10 min incubation at 70°C. At the end of the incubation, one volume of 100% ethanol was added to each sample, mixed, and transferred to HiBind® DNA Mini columns and centrifuged at 10,000 ×*g* for 1 min. DNA bound to columns was washed once with 500 μL of HB buffer and twice with 700 μL of DNA wash buffer followed by centrifugation step at 15,000 ×*g* for 2 min to remove residual wash solution. DNA was eluted by adding 100 μL of the Elution Buffer (preheated to 70°C) directly onto the HiBind® column matrix, and centrifugation at 10,000 ×*g* for 1 min after 2 min incubation at room temperature.

The nine microsatellites makers Mp13, Mp24, Mp26, Mp27, Mp29, Mp33, Mp34, Mp42, and Mp54 developed by Sanchez, La-Spina, and Perera were used in the genetic analyses (Molecular Ecology Resources Primer Development Consortium et al. 2011). Mp13, Mp24, Mp26, Mp29, Mp34, and Mp54 were dinucleotid repeats; Mp27 and Mp42 were trinucleotids, and Mp33 was a tretranucleotid repeat (Molecular Ecology Resources Primer Development Consortium et al. 2011). Amplification reactions were prepared using 2 μL of approximately 0.5 ng/μL of genomic DNA, 0.5 μM of each primer, 0.2 μM of dNTPs, 10 mM Tris HCl (pH 8.8), 50 mM KCl, 1.5 mM MgCl_2_, 0.01% of Tween and 0.5 U of DNA polymerase (DFS-Taq DNA polymerase, Bioron GmbH, Ludwigshafen, Germany), in a total volume of 20 μL. Forward primers were 5' labeled with 6-FAM, VIC, NED, or PET dyes (Applied Biosystems, Foster City, California). PCR amplifications were carried out with an Eppendorf Mastercycler EPgradient (Eppendorf AG, Hamburg, Germany) under the following conditions: 2 min at 94°C followed by 36 cycles of 15 sec at 94°C, 30 sec at 61°C, and 30 sec at 72°C, and then a 10 min incubation at 72°C. The PCR products were run as 1:50 dilution on an AB3730 DNA Analyzer (Applied Biosystems) with ROX-labeled size standard (Applied Biosystems). Fragments were detected with Peak Scanner™ software v1.0 (Applied Biosystems) and verified manually. Three positive and three negative controls were included on each 96-well PCR plate and some individuals from each population were scored twice.

### Statistical Genetic analyses

**Figure 2:**
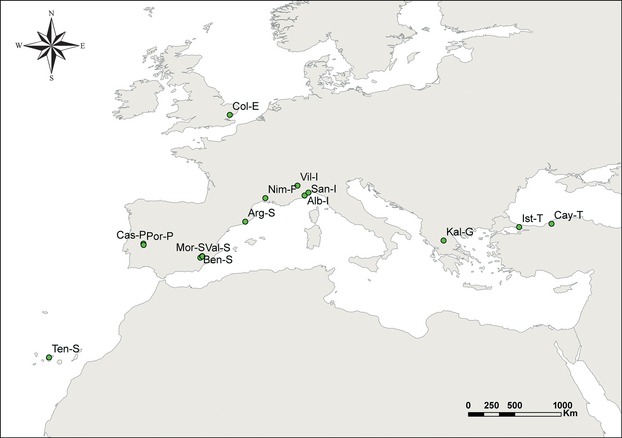
Map showing the distribution of *Macrolophus pygmaeus* populations used in the genetic analyses.

The number of individuals genotyped for each population is indicated in Table 1. The number of allele per locus and population, and the observed (*H*_*o*_) and expected (*H*_*e*_) heterozigosity were calculated using the FSTAT 2.9.3.2 software (Goudet 2002). The allelic richness was calculated by rarefaction using the HP-Rare software (Kalinowski 2004, 2005). The effective number of alleles (Ae) was calculated using GENALEX 6 (Peakall and Smouse 2006). The correlation between the allelic richness and *H*_*e*_ in function of the geographic distances to the most western locality (Tenerife, Canary Islands) was analyzed by the Pearson's correlation test; the sample from the commercial producer was not included in the analyses. Deviation from Hardy–Weinberg equilibrium (*HWE*), fixation index (*F*_*IS*_) values, and Genotypic linkage disequilibrium (*LD*) was calculated for each population using FSTAT (Goudet 2002). A Bonferroni correction (Rice 1989) for multiple testing was applied to all probabilities. The frequency of null alleles was calculated using Genepop 4.0 (Rousset 2008). Pairwise genetic distances for populations were calculated using Cavalli-Sforza and Edwards chord distance (*Dc*) (Cavalli-Sforza and Edwards 1967) using FSTAT (Goudet 2002); *Dc* is considered the most efficient distances in obtaining the correct tree topology under different conditions for microsatellite markers (Takezaki and Nei 1996). Besides, *Dc* is less affected by the presence of null alleles (Chapuis and Estoup 2007). An UPGMA dendrogram was constructed using the Population 1.2.32 software (Langella 1999). The consistency of the nodes was assessed by 1000 pseudo-replicates of the original data using the bootstrap method. The dendrogram was displayed using the Treeview software (Page 1996). The level of genetic differentiation between populations was quantitated by pairwise F_ST_ values according to Weir and Cockerham (1984), and *P*-values were obtained after 36000 permutations using the FSTAT software (Goudet 2002); probabilities were Bonferroni corrected (Rice 1989). The freeNA software was used to calculate F_ST_ values with the excluding null allele (ENA) correction method (Chapuis and Estoup 2007). Pairwise F_ST_ values were also calculated by pooling in the same population all the individuals from the same geographic area [The Canary Islands, the Iberian Peninsula, England, France, Italy and Greece-Turkey (Fig. 2, Table 1)]

To test the hypothesis of isolation by distance, the correlation between F_ST_/(1−F_ST_) and the geographic distances was calculated with the Mantel's test using the R software (R Development Core Team 2008). The analysis of molecular variance (AMOVA) was performed to determine the amount of genetic variability due to the main geographic areas using the ARLEQUIN 2.0 software (Excoffier et al. 2005). The sample from the commercial producer was not included in these analysis. The significance of the variance components was tested with 10,000 permutations. Principal Component Analysis (PCA) was performed to summarize the genetic variability on the microsatellite data set using the R packages *adegenet* and *ade4* (Chessel 2004; Jombart 2008). PCA has been considered very appropriate to study systems with little prior information because it does not assume Hardy–Weinberg equilibrium or linkage disequilibrium (Manel et al. 2003; Jombart 2008). The inter-class PCA, a modification of PCA maximizing the variance between populations, was used to determine the divergences among *M. pygmaeus* populations (Jombart 2008). The potential structure of the populations was analyzed from a Bayesian approach using the software STRUCTURE version 2.3.3 (Pritchard et al. 2000). The program was run 10 times for each K value using the admixture model with a burn-in period of 100,000 iterations and then 100,000 iterations of a Markov chain Monte Carlo from K equals 1–11. Prior information on sampling locations was provided. The *ad hoc* statistic ΔK, based on the rate of change in the log probability between successive K values, was used to detect the true numbers of clusters (K) (Evanno et al. 2005).

## Results

### Microsatellite Markers, linkage disequilibrium, and genetic diversity

**Figure 3:**
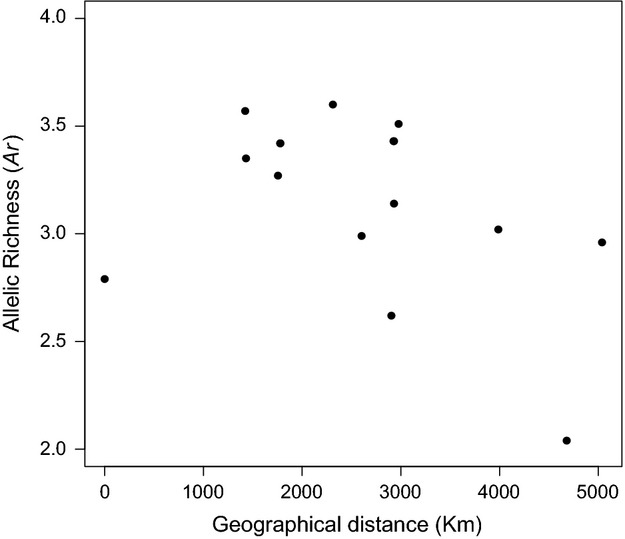
Allelic richness *versus* the distances in Km of each population to the farthest east locality (Ten-S: Tenerife, Canary Islands) in *Macrolophus pygmaeus*.

From the 367 individuals analyzed, 357 were unique genotypes. In the population from Caycuma (Turkey), two and four individuals shared two genotypes; and in the population from Istanbul (Turkey), two genotypes were found twice. The average number of alleles per locus per population was 5.5, ranging from 3.1 to 7.8 alleles per locus. The effective number of alleles ranged from 1.4 to 3.8 (average: 3.1 allele per locus per population), and allelic richness from 2.0 to 3.6 (average: 3.2 allele per locus per population) (Table S1). A significant correlation was found between the allelic richness and the geographic distance of each population to the western-most locality (Ten-S, Canary Islands) (Fig. 3) (Pearson correlation test, *r* = −0.661, t = −3.17, df = 12, *P* = 0.007), once Ten-S was excluded. The maximum number of private alleles was found at Val-S (8) and Cas-P (6), followed by Cay-T (4), Por-P (3), Kal-T (3); the remaining of the localities had less than 2 private alleles (Table S1).

The average *H*_*o*_ was 0.467, ranging from 0.237 in Ist-T to 0.567 in Arg-S. The average *H*_*e*_ was 0.539, ranging from 0.291 to 0.626 in Ist-T and Arg-S, respectively (Table S1). There was a highly significant correlation between *H*_*e*_ and the geographic distances (Pearson correlation test, *r* = −0.593, t = −2.65, df = 13, *P* < 0.05). Heterozygosity deficit, with significant departure from *HWE*, after the sequential Bonferroni correction, was observed at Mp24 in the Ben-S and Kal-G populations, Mp26 (Por-P), Mp34 (Cas-P, Col-E, Alb-I, KaL-G and Cay-T), and Mp54 (Cay-T) (Table S1). The average percentage of null alleles in loci across populations ranged from 1.1% in Mp27 to 12.6% in Mp34. The average percentage of null allele in populations across loci ranged from 2.3% in Ist-T population to 8.2% in Vil-I. Significant linkage disequilibrium, after Bonferroni correction, was observed between several of the loci in Cay-T (Mp13-Mp24, Mp13-Mp34, Mp13-Mp54, Mp24-Mp34, Mp27-Mp34, Mp34-Mp42, Mp34-Mp54, Mp42-Mp54) and once in Kal-G (Mp34-Mp54). The populations with significant heterozigosity deficit were generally those with a high proportion of null alleles.

### Population structure in *M. pygmaeus*

The pairwise F_ST_ values estimated using the ENA and the conventional method were very close in most of the cases and, although F_ST_ values were generally a little higher than F_ST_-ENA, there was no increase in the bias at high F_ST_ values (Figure S1). The F_ST_ values ranged from 0.000 to 0.334 and showed a high differentiation in *M. pygmaeus* populations through its geographic area of distribution. The matrix of pairwise F_ST_ values is given in Table 2. The highest F_ST_ values were found in populations from Istanbul *versus* those from Portugal (Cas-P, 0.334) and southern Spain (Mor-S, 0.331) (Table 2). The lowest F_ST_ values were found between Kal-G and Cay-T (0.000), between the two populations from Portugal (0.002), and between populations in southern Spain (0.004) (Table 2). The average of F_ST_ values in the Iberian Peninsula was 0.017 (0.002–0.044); the highest F_ST_ values in the Iberian Peninsula were found between the Arg-S and the rest of the populations (Table 2). Pairwise F_ST_ values between all populations from southern Spain and Portugal were low and not significantly different from zero. In Italy, there were no significant differences in pairwise F_ST_ values (Table 2). The F_ST_ value between the two populations from Turkey indicated a moderate population differentiation, but not significant; no differences were found between the Greek and Turkish populations (Table 2). The population from south of France was significantly different from the rest of the populations, with the exception of the commercial strain. The *M. pygmaeus* population in southern England differed significantly from all the others (Table 2). Pairwise F_ST_ values pooling all the individuals from the same geographic area in the same population reflected the results of the analyses using the single populations (Table 3). Significant differences were found between populations in the main geographic areas, with the exception of the Greek and Turkish populations (Table 3). The hypothesis of isolation of *M. pygmaeus* by distances was supported by a high positive correlation between the genetic divergence in populations, expressed as F_ST_/(1−F_ST_), and the geographic distances (Mantel statistic, *r* = 0.7453, *P* < 0.001). In the AMOVA analysis, most of the genetic variations were due to differences between individuals within populations (84.18%) (Table 4). However, a significant percentage of the variation (14.27%) was explained by differences among geographic areas (England, Greece-Turkey, Iberia, Italy, France and the Canary Islands), and among populations within areas (1.55%).

**Table 2:** Pairwise comparison for genetic differentiation between populations based on F_ST_ for *Macrolophus pygmaeus*

	Canary I.	Portugal		Spain				UK	France	Italy			Greece	Turkey		
									
	Ten-S	Cas-P	Por-P	Arg-S	Ben-S	Mor-S	Val-S	Col-E	Nim-F	Alb-I	San-I	Vil-I	Kal-G	Cay-T	Ist-T	C
Ten-S		NS	*	**	*	NS	NS	**	***	***	**	**	*	**	**	***
Cas-P	0.071		NS	NS	NS	NS	NS	***	***	***	***	***	***	***	***	***
Por-P	0.078	0.002		NS	NS	NS	NS	***	***	***	***	***	***	***	***	***
Arg-S	0.082	0.044	0.031		**	NS	*	***	***	NS	NS	**	***	***	***	***
Ben-S	0.080	0.007	0.009	0.035		NS	NS	***	***	***	***	***	***	***	***	***
Mor-S	0.069	0.005	0.004	0.041	0.004		NS	***	***	***	***	***	***	***	***	***
Val-S	0.065	0.003	0.018	0.042	0.002	0.008		***	***	***	***	***	***	***	***	***
Col-E	0.121	0.109	0.102	0.052	0.084	0.108	0.092		***	***	***	***	***	***	***	***
Nim-F	0.201	0.191	0.171	0.099	0.160	0.191	0.165	0.129		***	***	***	***	***	***	NS
Alb-I	0.150	0.145	0.119	0.023	0.122	0.137	0.133	0.095	0.076		NS	NS	***	***	***	***
San-I	0.101	0.119	0.091	0.027	0.094	0.103	0.100	0.073	0.070	0.013		NS	***	***	***	***
Vil-I	0.155	0.155	0.129	0.039	0.134	0.154	0.139	0.077	0.056	0.009	0.015		***	***	***	***
Kal-G	0.164	0.213	0.206	0.161	0.178	0.196	0.178	0.174	0.159	0.187	0.118	0.166		NS	NS	***
Cay-T	0.185	0.236	0.232	0.201	0.194	0.212	0.198	0.210	0.206	0.230	0.148	0.220	0.000		NS	***
Ist-T	0.282	0.334	0.314	0.284	0.282	0.331	0.297	0.286	0.299	0.302	0.224	0.281	0.060	0.056		***
C	0.230	0.210	0.193	0.124	0.176	0.224	0.181	0.149	0.005	0.098	0.094	0.059	0.179	0.224	0.319	

NS= non-significant.

* *P* < 0.05.

** *P* < 0.01.

*** *P* < 0.001.

Probabilities corrected from Bonferroni.

**Table 3:** Pairwise comparison for genetic differentiation between geographic areas based on F_ST_ for *Macrolophus pygmaeus*

	Canary I.	Iberia	UK	France	Italy	Greece	Turkey
Canary I.		*	***	**	***	**	***
Iberia	0.067		***	***	***	***	***
UK	0.121	0.083		***	***	***	***
France	0.198	0.151	0.127		***	***	***
Italy	0.128	0.102	0.076	0.058		***	***
Greece	0.141	0.150	0.153	0.133	0.130		NS
Turkey	0.152	0.157	0.176	0.182	0.161	0.007	

NS= non-significant.

* *P* < 0.05.

** *P* < 0.01.

*** *P* < 0.001.

Probabilities corrected from Bonferroni.

**Table 4:** Results of the hierarchical Analysis of molecular variance AMOVA for 15 populations of *Macrolophus pygmaeus*

Source of variation	df	SSD	Variance Component	Variation Percentage	*P*-value
Among groups	5	227.5	0.3905	14.27	<0.001
Among populations within groups	10	43.3	0.0425	1.55	<0.001
Within populations	718	1654.1	2.3037	84.18	<0.001

Percentage of the total genetic variance due to each level and the probability test after 10000 permutations. Degrees of freedom (df), sums of square deviations (SSD).

**Figure 4:**
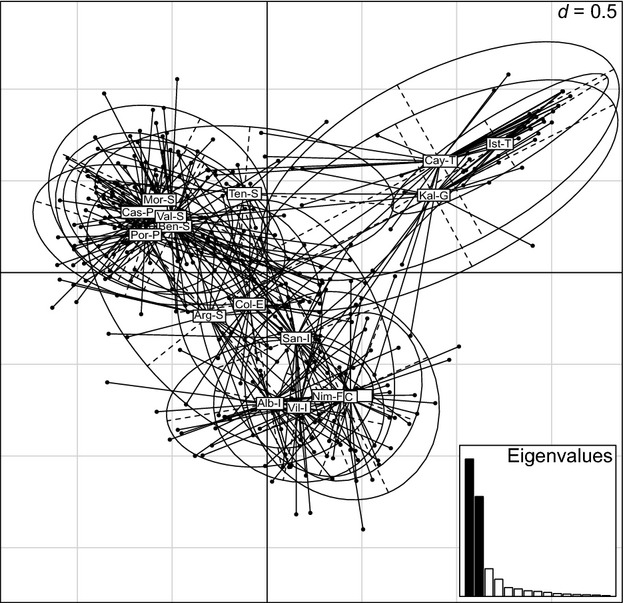
Topology of *Macrolophus pygmaeus* from different localities in the Palaearctic region obtained by Principal Component Analysis (PCA) using microsatellite markers (Population codes in Table 1). Points are genotypes; populations are labeled inside their 95% inertia ellipses.

**Figure 5:**
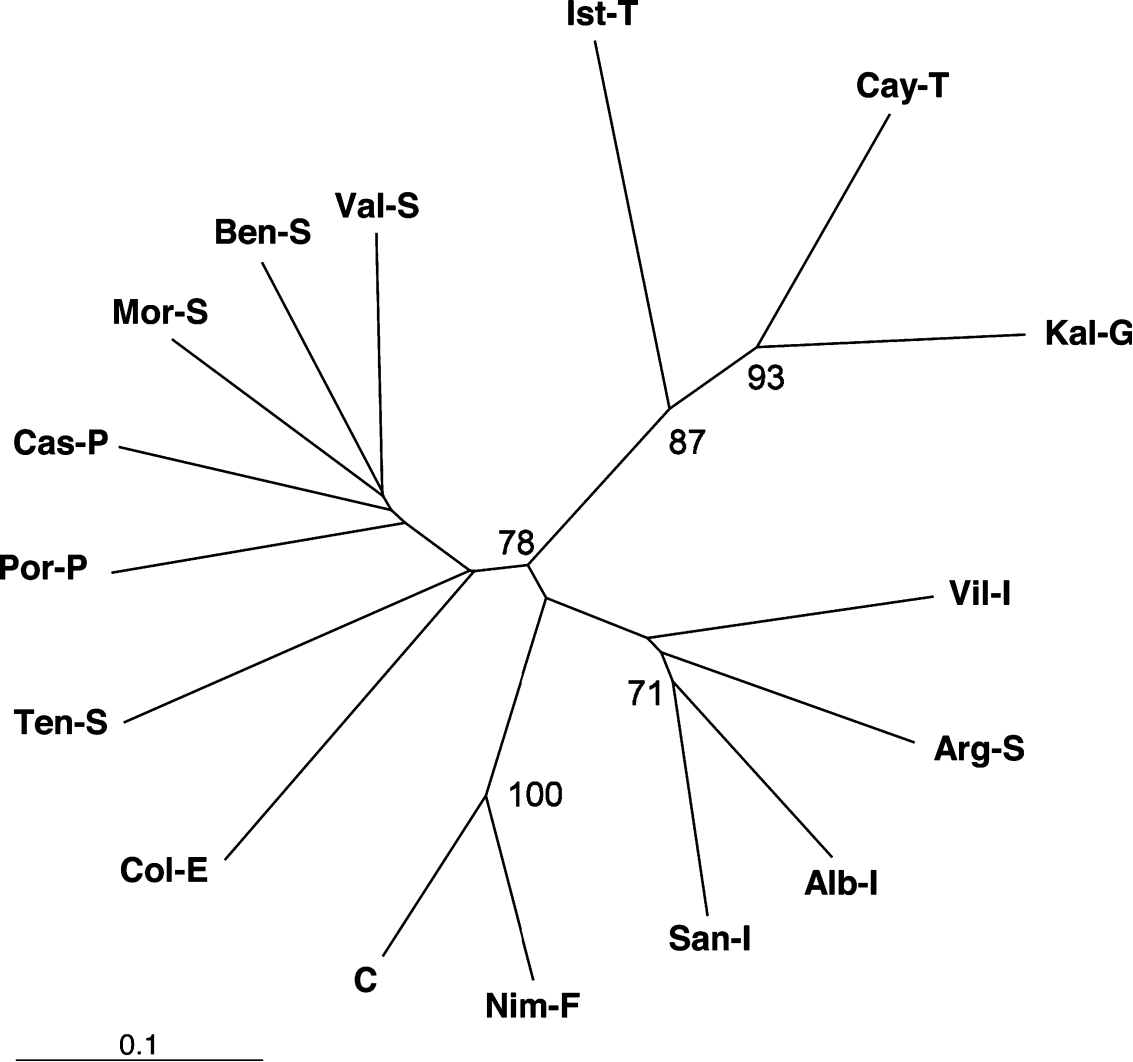
UPGM dendrogram constructed after 100 bootstrap re-sampling based on Cavalli-Sforza and Edwards chord distance (*D*_*C*_) (Cavalli-Sforza and Edwards 1967) showing the relationship among populations of *Macrolophus pygmaeus* in the Palaearctic region (Population codes in Table 1).

In the PCA, the first two axes explained 31.5 and 22.9% of the variance in the experimental data, respectively. Populations were clustered into groups based on geographic proximity, except for Col-E, which fell in between groups of the Iberian and the Italian–French populations (Fig. 4). The axis 1 separates the populations from southern Greece and Turkey from those of Italy and France and the axis 2 separates the populations from Iberia and the Canary Islands from those of Italy and Greece–Turkey (Fig. 4). The UPGMA distance tree was in agreement with the PCA analysis. The first main cluster was integrated by the Turkish and Greek populations, the second one with the populations from Portugal, southern Spain, the Canary Islands and England, and the third cluster with Arg-S, Italian and French populations (Fig. 5). The consistency of branching for the three main cluster in the tree dendrogram was supported by acceptable bootstrap values at nodes (Fig. 5).

**Figure 6:**
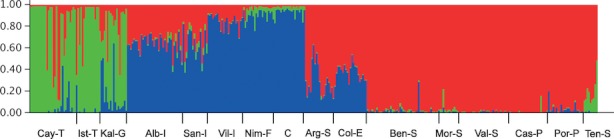
STRUCTURE analyses assuming K = 3 populations. *Macrolophus pygmaeus* population structure based on microsatellite scores. Each line represents a single individual and it is fragment in different colors according to the coefficient of ancestry of each individual. Populations are represented from East (right) to the West (left) (Fig. 2).

The STRUCTURE analysis was run for K values ranging from 1 to 11, with the *ad hoc* statistic ΔK reaching its maximum at K = 3. According to these analyses, the population of *M. pygmaeus* could be split in three main clusters. Figure 6 shows the values of the coefficients of ancestry for each *M. pygmaeus* individual. Moving from east to west, the following clusters may be observed in Figure 6: a first cluster integrated by the Turkish and Greece populations; a second one including the Italian and French populations, a transition zone integrated by the Arg-S and Col-E populations sharing a greater degree of ancestry with the Iberian than with the Italian–French populations; and the third cluster including the populations from Portugal and southern Spain. The population from the Canary Islands showed a great degree of ancestry with the populations of southern Iberia. *Macrolophus pygmaeus* had high average coefficients of ancestry (0.757–0.880) in cluster 3, with a high percentage of the individuals (81–94) being assigned to this cluster (Table 5). The coefficients of ancestry in cluster 3 for the Greek population (0.649) were lower than the Turkish's (0.757–0.880); most of *M. pygmaeus* from Greece were assigned to cluster 3, but some were also assigned to cluster 1 and 2 (Table 5). *Macrolophus pygmaeus* populations from the Liguria (Alb-S, San-I) in Italy showed a shared ancestry with the Italian–French and Iberian populations, with some of the individuals from Sanda assigned to the Iberian cluster (Table 5). Populations from the northern interior of Italy (Vil-I) showed a higher degree of Ancestry with the Italian–French cluster, with all the individuals in the population belonging to this group. Arg-S and Col-E populations had a share ancestry with Iberian and Italian–French cluster, but some of the individuals were included in the Italian–French cluster. The *M. pygmaeus* from Portugal, southern Spain, and the Tenerife in the Canary Islands had high coefficients of ancestry and assignment in the Iberian cluster (Table 5).

**Table 5:** Average coefficients of ancestry obtained in Bayesian analysis using STRUCTURE with K = 3 for 367 *Macrolophus pygmaeus* collected in the Palaearctic region

Geographic area	Population	Cluster 1	*N*	Cluster 2	*N*	Cluster 3	*N*
Canary Islands	Ten-S	0.803	94	0.014	6	0.183	0
Portugal	Cas-P	0.967	100	0.012	0	0.021	0
	Por-P	0.939	100	0.055	0	0.006	0
Spain	Arg-S	0.684	81	0.308	20	0.008	0
	Ben-S	0.955	100	0.031	0	0.015	0
	Mor-S	0.943	100	0.021	0	0.037	0
	Val-S	0.977	100	0.018	0	0.006	0
United Kingdom	Col-E	0.650	97	0.346	4	0.005	0
France	Nim-F	0.031	0	0.895	100	0.074	0
Italy	Alb-I	0.333	0	0.660	100	0.006	0
	San-I	0.329	12	0.608	88	0.063	0
	Vil-I	0.150	0	0.845	100	0.006	0
Greece	Kal-G	0.189	13	0.162	12	0.649	75
Turkey	Cay-T	0.191	19	0.052	0	0.757	81
Turkey	Ist-T	0.106	6	0.014	0	0.880	94
Commercial strain	C	0.014	0	0.930	100	0.057	0

*N*, percentage of individuals in the populations assigned to the cluster.

## Discussion

A genetic population analysis was performed for the plant bug *M. pygmaeus* throughout a wide range of its geographic distribution using nine microsatellite markers. Samples of *M. pygmaeus* were collected on *S. lycopersicon* in 15 localities (Fig. 2), and a sample from a commercial producer was also included in the analyses. The moderate number of alleles per locus (5.5 alleles per locus per population) showed the utility of these molecular markers for population studies in *M. pygmaeus*. The average number of alleles per locus in *M. pygmaeus* was lower than that found in other mirid species such as *Lygus lineolaris* (Palisot de Beauvois) (Hemiptera: Miridae) (12.25 alleles per locus) (Perera et al. 2007) and *Stenotus rubrovittatus* (Matsumura) (Hemiptera: Miridae) (11.4 alleles per locus) (Kobayashi et al. 2011). *Lygus heperus* Knight (Hemiptera: Miridae) had similar numbers (4.5 alleles per locus) than *M. pygmaeus* (Shrestha et al. 2007). Significant linkage disequilibrium was observed among several loci in one of the Turkish and the Greek population. Inbreeding, small population size, genetic isolation between lineages, mixing of populations during sampling, and population subdivision are some of the factors that may lead to linkage disequilibrium (Rafalski and Morgante 2004; Gupta et al. 2005). The variation in linkage disequilibrium has been also associated with bottlenecks (Tishkoff et al. 1996). In the case of *M. pygmaeus,* the linkage in the populations from Turkey and Greece might be due to isolation and bottlenecks. This is in agreement with the low genetic diversity and the low allelic richness in these two geographic areas, and to the presence of individuals with the same score for the nine microsatellite loci. Population bottlenecks may have resulted in the loss of alleles and allele diversity may have not been compensated with the migration of individuals from other areas. The presence of individuals with the same score for the nine microsatellites might be explained by the low genetic diversity, the founder effect, and the high reproduction rates of this mirid species. Tomatoes are temporal plants where *M. pygmaus* populations have to re-establish on every yearly cycle; populations may arise from very few founder adults and many individuals in a population may come from the same progenitors in the initial growing phase. Linkage disequilibrium due to inbreeding is excluded because it would produce significant departure from HWE in most of the loci (Schlötterer 1998), and in our case, heterozygosity deficit happens only in a few of them. The heterozygosity deficit detected in a few of the loci could be most likely due to null alleles. The high diversity in the Italian and Iberian Peninsula suggest that *M. pygmaeus* in these areas did not suffer from the bottlenecks of the Balkans and Asia Minor. The lower diversity in the Canary Islands may be a consequence of the loss of diversity in the process of colonization of the islands from the mainland. The fixation of two of the loci (Mp27 and Mp33) is also symptoms of a bottleneck. Seabra et al. (2009) argued that the lower microsatellite variability and heterozygosity of *Cicada orni* L. (Hemiptera: Cicadidae) in the Aegean islands compared with mainland Greek was possibly due to a reduced gene flow and bottlenecks during the process of colonization of the islands. When just a small numbers of individuals takes part in the colonization of new areas, the genetic variability of the founder population is expected to be lower than the original one (Zygouridis et al. 2009).

Bayesian analyses, PCA, and UPGMA analyses indicates the existence of three main population clusters in *M. pygmaeus*: one cluster including the populations from Greece and Turkey; a second cluster including the populations from Italy and France; and a third cluster with most of the populations from the Iberian Peninsula and the Canary Islands. The consistency of nodes for the three main clusters was supported by acceptable bootstrap values in the UPGMA analyses; the *ad hoc* statistic (ΔK), used to determine the true number of populations using the software STRUCTURE, reached its maximum value at K = 3. Structure is not considered as suitable to describe relationships among population, but it produces the appropriate number of evolutionary cluster under many circumstances (Kalinowski 2011). AMOVA analyses also denoted a significant contribution of the difference between geographic areas to the genetic variability of the species. *Macrolophus pygmaeus* showed a moderate to high degree of population differentiation among the three main geographic areas of distribution, with the highest degree of divergence found between eastern (Turkey and Greece) and western populations (Italy, France, England, Iberia, and the Canary Islands) (F_ST_ values= 0.334–0.194). This high degree of population differentiation in the main peninsulas could be explained by the isolation of *M. pygmaeus* in these areas during long periods of time. Finding the highest number of private alleles in southern Iberia and Turkey is in agreement with the hypothesis of the old colonization of these two areas. Many species of animals and plants have evolved into different genetic lineages in the three Mediterranean peninsulas during periods of isolations in the glaciations (Hewitt 1999, 2000; Taberlet et al. 1998). Habel et al. (2005) concluded that the *Melanargia galathea* (L.)/*Melanargia lachesis* (Hübner) (Satyrinae: Lepidoptera) sibling species complex represented three major genetic lineages, each originating from one of the three major refugial areas in southern Europe. Schmitt and Seitz (2001) argued that the differentiation (F_ST_ values = 0.060) in the widely distributed and abundant butterfly *Polyommatus coridon* (Poda) (Lepidoptera: Lycaenidae) was best explained by the long disjunction in two ice-age refugia of the Adriato-Mediterranean (peninsular Italy) and Ponto-Mediterranean (the Balkan Peninsula, Asia Minor and the east Mediterranean coast). In the same way, *Maniola jurtina* (L.) (Lepidoptera: Nymphalidae) shows two major genetic lineages: the Atlantico-Mediterranean and the Adriatic-Pontic-Mediterranean subpopulations (Schmitt et al. 2005; Schmitt 2007). Three subpopulations were detected in *B. oleae* from the Western Mediterranean (Spain and Portugal), Central Mediterranean (Greece, Italy, and, possibly, Turkey), and Eastern Mediterranean (Cyprus) (Augustinos et al. 2005; Zygouridis et al. 2009). Zygouridis et al. (2009) found that populations from Greece, Italy, and possibly Turkey form a distinct subpopulation, likely due to extensive gene flow in the area, which may be attributed to the historical trade between the areas. In contrast, this study on *M. pygmaeus* is one of the few cases reporting high divergences in the subpopulations of a species between the Italian and the Balkan Peninsulas. To our knowledge, differentiations between the lineages in the Italian and the Balkan Peninsula have been reported only in *Fagus sylvatica* L. (Fagaceae) (Demesure et al. 1996). In other species of insects, such as the grasshopper *Chorthippus parallelus* (Zetterstedt) (Orthoptera: Acrididae) (Cooper et al. 1995) and the bark beetle *Tomicus destruens* Woll. (Coleoptera: Scolytinae) (Horn et al. 2006, 2009), some population differentiation was found between the Balkans and Asia Minor. In the case of *M. pygmaeus*, the Balkans and Asia Minor seem to represent a distinct population. The absence of differentiation between the populations from Portugal and southern Spain, which are about 600 km apart, indicates the existence of a continuous flow of individuals between remote localities in eastern and western Iberia. These movements would be facilitated by the absence of mountain chains between eastern and western Iberia. Hamarsheh et al. (2009) argued that genetic differentiation can be low, even between populations separated by many thousands of kilometers, unless there are major hydrographic or geographic barriers to migration. F_ST_ values (0.065–0.080) indicate a moderate population differentiation in *M. pygmaeus* between southern Iberia and the Canary Islands; the lack of significance and high variance in F_ST_ values between some populations in southern Iberia and the Canary Islands could be due to the low number of individuals in the Canary Island sample. The connection of *M. pygmaeus* subpopulations between the Iberian Peninsula and the Canary Islands was probably facilitated by the reduction in the Strait of Gibraltar and by the stepping-stone corridor of islands between the Canaries, Madeira, and the Iberian Peninsula in the interglacial periods during the Pleistocene (Whittaker and Fernández-Palacios 2007). Habel et al. (2008) reported the recent connection between subpopulations of the butterfly *M. galathea* in Iberia and northern Africa due to their genetic similarity. F_ST_ values (0.023–0.027) between the populations of *M. pygmaeus* in northern Spain (Argentona) and the Liguria coast in Italy were low and not significantly different from zero, which indicates null or low population differentiation between these two geographic areas. These two populations were also included in the same cluster in the UPGMA analysis. In contrast, F_ST_ values (0.099) between northern Spain and southern France indicated a moderate differentiation in *M. pygmaeus* populations. These results are intriguing because a higher similarity would be expected between population in northern Spain and southern France due to their geographic proximity. If these were really what happened naturally, we would have to admit that the species is able to disperse through vast open seas, through a hypothetical bridge between northern Spain, Corsica or Sardinia, and the Liguria coast (Fig. 2), which seems quite unlikely. Another striking result is that the differentiation between the *M. pygmaeus* population in the Liguria (Italy) and northern Spain (F_ST_ values= 0.023–0.027) was lower than that between southern and northern populations in Spain (F_ST_ values= 0.035–0.042). If these results were to be explained by physical barriers interfering with the movement of the species, we would have to admit that the mountains in central Spain are a stronger physical barrier for the dispersal of the species than the Alps and the Pyrenees put together. The high degree of similarity between population of *M. pygmaeus* in northern Spain and Italy could be explained by: (1) selective environmental factors (e.g., similarities in climatic conditions between northeast coast in Spain and Liguria); (2) the ad-mixture of wild population and commercial strains native to the Italian peninsula in northern Spain. Zygouridis et al. (2009) argued that trade may be in part responsible for the current population structure of *B. oleae* in the Mediterranean basin. Besides, the possibility of human and environmental factors interfering with the distribution of populations in *M. pygmaeus* the high correlation between genetic differences among populations, and geographic distances also indicates a contribution of geographic distance to the genetic structure of *M. pygmaeus* populations. Augustinos et al. (2005) also found a strong correlation between genetic distances and geographic distances in *B. oleae*.

Based on the results of this study, we predict the recent history of *M. pygmaeus* as follows: (1) the reduction in the geographic distribution of the species to the Iberian, Italian, and Balkan peninsulas during the Pleistocene glaciations; the survival of the species in southern France is not excluded, and *M. pygmaeus* quite likely also survived in northern Africa. Konnert and Bergmann (1995) concluded that southeastern France was a glacial refuge for *Abies alba* Mill. (Pinaceae). (2) The maintenance of high populations and diversity of *M. pygmaeus* in Iberia, Italy, and possibly southern France, in the periods of contraction, with a reduction in population size and bottlenecks in the Balkans. (3) The introgression of the Italian–French lineage in northern Spain (naturally or through the trade of the species), and the likely expansion of the species from the Balkans to Asia Minor, given the similarities of subpopulations and the lower diversity in the latter area. Based on the genetic similarities of the British, the Iberian, and the Italian–French populations to northern European populations, it is possible that *M. pygmaeus* spread to northern Europe from its southern refuges in Iberia, Italy, and likely from the south of France.

This study proves that microsatellite could be used as powerful markers to determine the genetic structure of *M. pygmaeus* populations. The use of microsatellite markers may have a very practical approach in biological control, especially in conservation programs, as it would allow the identification of the source of the individuals colonizing crops. Microsatellite marker may also help to understand the introgression of commercial strains into wild populations. This study gives for the first time insight into the population structure of a mirid species in the Palaearctic region, which may also help to explain the current geographic distribution and speciation of other plant bugs in the area. The findings of this study provide a better understanding of the evolution and ecology of *M. pygmaeus* and could be used to study other mirid species with a similar biology. Divergences in ecological traits (e.g., host plant preference, degree of phytophagy) in *M. pygmaeus* subpopulations in the different geographic areas may also produce diverse outcomes in population dynamics and when using the species for pest control. More populations have to be analyzed to determine the population structure of *M. pygmaeus* in middle and northern Europe, and to understand the role of northern Africa as a refuge for the species during the glaciations and its connections with other geographic areas in the Middle East, Asia Minor, and the Balkans.

## Supporting Information

**Table S1.** Number of alleles (*N*), Ae (effective number of alleles), Ar (Allelic richnes), Pa (number of private alleles), observed (*H*_*o*_) and expected (*H*_*e*_) heterozygosity, and F_IS_ values for nine microsatellite loci in 16 populations of *M. pygmaeus*. Locality codes in Table 1. * Significant departure from HWE (*P* < 0.05), after correction from Bonferroni.

**Figure S1.** F_ST_ values calculated using the excluding null allele method correction method (F_ST_-ENA) versus Fst using the uncorrected data (F_ST_).
