# Supplementary material for: Analysis of the population structure of Macrolophus pygmaeus (Rambur) (Hemiptera: Miridae) in the Palaearctic region using microsatellite markers
Source: Ecol Evol. 2012 Nov 8;2(12):3145–59. doi: 10.1002/ece3.420 (PMC3539007; doi:10.1002/ece3.420)
Supplement: Supplementary file 1 [file ece30002-3145-SD4.pdf]

Locus		Canary I.	Portugal		Spain				UK	France	Italy			Greece	Turkey		
		Ten-S	Cas-P	Por-P	Arg-S	Ben-S	Mor-S	Val-S	Col-E	Nim-F	Alb-I	San-I	Vil-I	Kal-G	Cay-G	Ist-T	C
**Mp13**	**N**	5	10	13	11	15	7	20	9	6	13	12	10	7	9	4	3
	**Ae**	7.0	5.8	7.2	7.9	7.7	6.1	8.5	4.1	2.9	8.2	7.7	7.1	2.5	3.0	1.6	1.9
	**Ar**	4.5	5.2	5.6	5.7	5.6	5.2	6.1	4.6	3.3	5.8	5.5	5.2	3.5	3.9	2.3	2.2
	**Pa**	0	0	1	0	1	0	4	1	0	1	1	0	1	0	0	0
	**Ho**	0.571	0.833	0.920	0.750	0.769	0.846	0.765	0.800	0.818	0.962	0.808	0.917	0.682	0.642	0.267	0.550
	**He**	0.857	0.826	0.860	0.873	0.870	0.837	0.882	0.758	0.653	0.879	0.870	0.859	0.595	0.664	0.361	0.483
	**Fis**	0.314	-0.009	-0.071	0.130	0.117	-0.011	0.126	-0.057	-0.260	c	0.067	-0.093	-0.150	0.019	0.132	-0.142
**Mp24**	**N**	3	3	2	2	4	3	6	3	3	2	2	3	5	7	2	3
	**Ae**	3.4	1.6	1.4	2.3	1.9	1.5	2.3	1.9	1.7	1.5	1.6	1.4	1.6	2.0	1.2	1.5
	**Ar**	3.0	2.1	1.8	2.0	2.3	2.1	3.0	2.2	2.4	1.8	1.9	1.9	2.5	2.9	1.3	2.1
	**Pa**	0	1	0	0	0	0	1	0	0	0	0	1	0	1	0	0
	**Ho**	0.143	0.125	0.040	0.250	0.133	0.154	0.441	0.550	0.364	0.269	0.083	0.292	0.059	0.340	0.067	0.400
	**He**	0.703	0.363	0.285	0.560	0.460	0.345	0.567	0.479	0.409	0.324	0.378	0.295	0.374	0.495	0.131	0.337
	**Fis**	0.800	0.619	0.840	0.518	0.698*	0.461	0.197	-0.244	0.113	0.079	0.757	-0.126	0.822*	0.291	0.000	-0.192
**Mp26**	**N**	2	6	8	6	6	5	5	3	5	5	4	5	3	2	2	3
	**Ae**	1.6	1.6	2.3	3.5	1.7	2.3	1.3	1.6	2.8	2.2	2.5	2.7	1.3	1.2	1.2	2.5
	**Ar**	2.0	2.4	3.4	3.8	2.7	3.5	1.9	2.3	3.2	2.9	2.6	2.9	1.8	1.6	1.3	2.6
	**Pa**	0	1	0	0	0	1	0	0	1	0	0	0	0	0	0	0
	**Ho**	0.222	0.280	0.280	0.800	0.392	0.600	0.152	0.300	0.700	0.630	0.423	0.625	0.238	0.115	0.067	0.550
	**He**	0.366	0.383	0.563	0.713	0.417	0.568	0.228	0.392	0.647	0.549	0.597	0.635	0.220	0.193	0.131	0.596
	**Fis**	0.407	0.206	0.507*	-0.126	0.060	-0.059	0.247	0.140	-0.086	-0.151	0.272	-0.010	-0.087	0.345	0.000	0.032
**Mp27**	**N**	1	3	2	4	4	2	2	2	1	3	4	3	2	2	1	1
	**Ae**	NA	1.2	1.4	1.3	1.4	1.7	1.3	1.1	NA	1.5	1.6	1.2	1.2	1.4		NA
	**Ar**	1.0	1.5	1.8	1.9	2.1	1.9	1.7	1.2	1.0	2.1	2.4	1.8	1.4	1.8	1.0	1.0
	**Pa**	0	0	0	0	1	0	0	0	0	0	0	0	0	0	0	0
	**Ho**	NA	0.125	0.292	0.200	0.314	0.231	0.235	0.050	NA	0.222	0.385	0.208	0.111	0.282	NA	NA
	**He**	NA	0.160	0.290	0.236	0.296	0.397	0.237	0.099	NA	0.328	0.372	0.194	0.160	0.267	NA	NA
	**Fis**	NA	-0.030	-0.150	-0.048	-0.123	0.294	-0.119	0.000	NA	0.255	-0.131	-0.075	-0.030	-0.152	NA	NA
**Mp29**	**N**	4	7	7	7	8	7	7	6	5	6	7	6	8	5	5	3
	**Ae**	3.2	5.2	3.8	3.1	3.2	4.9	3.0	4.2	2.6	2.9	4.5	2.9	3.9	2.8	1.8	2.4
	**Ar**	3.5	4.4	4.1	3.9	3.6	4.7	3.6	4.2	3.0	3.7	4.2	3.8	4.3	3.2	2.9	2.5
	**Pa**	0	0	2	0	0	0	1	0	0	0	1	0	1	0	0	0
	**Ho**	0.556	0.739	0.720	0.700	0.745	0.750	0.469	0.800	0.550	0.444	0.654	0.375	0.591	0.462	0.400	0.550
	**He**	0.686	0.808	0.739	0.678	0.691	0.797	0.668	0.764	0.612	0.649	0.779	0.655	0.745	0.641	0.455	0.581
	**Fis**	0.200	0.060	0.013	-0.033	-0.096	0.062	0.301	-0.048	0.103	0.320	0.148	0.427	0.211	0.281	0.120	0.054
**Mp33**	**N**	1	5	5	4	3	3	6	3	5	5	3	4	4	4	2	3
	**Ae**	NA	1.5	1.5	1.4	1.3	1.3	1.3	1.6	1.5	1.4	1.4	1.5	1.5	1.2	1.3	1.2
	**Ar**	1.0	2.3	2.4	2.0	1.7	1.8	1.9	2.1	2.2	2.2	1.9	2.2	2.1	1.7	1.8	1.6
	**Pa**	0	2	0	0	0	0	1	0	1	0	0	0	1	1	0	0
	**Ho**	NA	0.120	0.280	0.250	0.118	0.167	0.206	0.400	0.227	0.333	0.308	0.250	0.318	0.176	0.267	0.150
	**He**	NA	0.327	0.325	0.278	0.202	0.239	0.222	0.377	0.328	0.298	0.271	0.337	0.323	0.166	0.239	0.191
	**Fis**	NA	0.593	0.138	-0.067	0.358	-0.023	-0.052	-0.192	0.219	-0.122	-0.140	0.166	-0.118	-0.061	-0.120	-0.036
**Mp34**	**N**	5	12	13	10	16	9	12	7	6	11	11	9	7	13	5	8
	**Ae**	6.3	6.1	6.6	7.5	5.9	6.7	6.7	5.9	5.7	5.8	5.6	5.9	3.5	5.0	2.3	5.0
	**Ar**	4.1	5.4	5.4	5.6	5.4	5.2	5.4	4.5	4.6	5.1	5.2	5.2	4.0	5.0	3.0	4.9
	**Pa**	0	0	0	0	0	0	0	0	0	0	0	0	0	2	0	0
	**Ho**	0.625	0.520	0.667	0.650	0.787	0.667	0.676	0.450	0.550	0.519	0.577	0.565	0.050	0.500	0.533	0.632
	**He**	0.842	0.836	0.848	0.867	0.831	0.851	0.850	0.831	0.824	0.827	0.821	0.830	0.710	0.799	0.572	0.799
	**Fis**	0.195	0.366*	0.205	0.250	0.053	0.204	0.191	0.451*	0.325	0.368*	0.287	0.321	0.927*	0.366*	-0.014	0.215
**Mp42**	**N**	2	3	5	4	3	2	4	4	4	4	4	6	5	4	2	4
	**Ae**	2.0	2.2	2.2	2.8	1.9	1.6	2.4	3.6	3.5	3.0	2.4	3.9	2.2	1.6	1.2	2.9
	**Ar**	2.0	2.2	2.5	3.1	2.1	1.9	2.6	3.6	3.4	3.0	2.7	3.8	3.1	2.3	1.3	3.2
	**Pa**	0	0	0	0	0	0	1	0	0	0	0	1	0	0	0	0
	**Ho**	0.778	0.542	0.680	0.600	0.563	0.385	0.824	0.750	0.864	0.704	0.615	0.708	0.650	0.396	0.067	0.800
	**He**	0.503	0.537	0.544	0.638	0.463	0.385	0.582	0.719	0.714	0.662	0.575	0.746	0.545	0.359	0.131	0.658
	**Fis**	-0.600	-0.042	-0.257	0.060	-0.218	-0.200	-0.451	-0.056	-0.253	-0.086	-0.071	0.035	-0.199	-0.158	0.000	-0.223
**Mp54**	**N**	5	10	8	6	8	6	8	4	5	6	9	6	9	11	6	5
	**Ae**	4.4	4.6	6.5	4.9	4.0	4.9	4.5	3.4	4.0	4.0	6.2	4.3	3.7	4.3	2.5	2.7
	**Ar**	4.1	4.6	5.2	4.5	3.9	4.5	4.6	3.5	3.8	4.1	5.0	4.1	4.4	4.4	3.5	3.3
	**Pa**	0	2	0	0	0	0	0	0	0	0	0	0	0	0	0	0
	**Ho**	0.778	0.520	0.750	0.900	0.712	0.846	0.765	0.850	0.636	0.630	0.792	0.652	0.526	0.288	0.467	0.600
	**He**	0.771	0.781	0.847	0.795	0.748	0.797	0.778	0.704	0.752	0.753	0.838	0.765	0.730	0.768	0.595	0.628
	**Fis**	-0.009	0.322	0.115	-0.138	0.033	-0.065	0.017	-0.214	0.142	0.156	0.054	0.151	0.255	0.620*	0.152	-0.002
**Average**	**N**	3.1	6.6	7.0	6.0	7.4	4.9	7.8	4.6	4.4	6.1	6.2	5.8	5.6	6.3	3.2	3.7
	**Ae**	3.1	3.3	3.7	3.8	3.2	3.5	3.5	3.1	2.7	3.4	3.7	3.4	2.4	2.5	1.4	2.2
	**Ar**	2.8	3.4	3.6	3.6	3.3	3.4	3.4	3.1	3.0	3.4	3.5	3.4	3.0	3.0	2.0	2.6
	**Pa**	0.0	0.7	0.3	0.0	0.2	0.1	0.9	0.1	0.2	0.1	0.2	0.2	0.3	0.4	0.0	0.0
	**Ho**	0.408	0.423	0.514	0.567	0.504	0.516	0.504	0.550	0.523	0.524	0.516	0.510	0.358	0.356	0.237	0.470
	**He**	0.525	0.558	0.589	0.626	0.553	0.580	0.557	0.569	0.549	0.585	0.611	0.591	0.489	0.484	0.291	0.475
	**Fis**	0.145	0.232	0.149	0.061	0.098	0.074	0.051	-0.024	0.034	0.080	0.138	0.088	0.181	0.172	0.030	-0.033

Supplementary material - Table 1
