# Supplementary figures and images for: Analysis of the population structure of Macrolophus pygmaeus (Rambur) (Hemiptera: Miridae) in the Palaearctic region using microsatellite markers
Source: Ecol Evol. 2012 Nov 8;2(12):3145–59. doi: 10.1002/ece3.420 (PMC3539007; doi:10.1002/ece3.420)

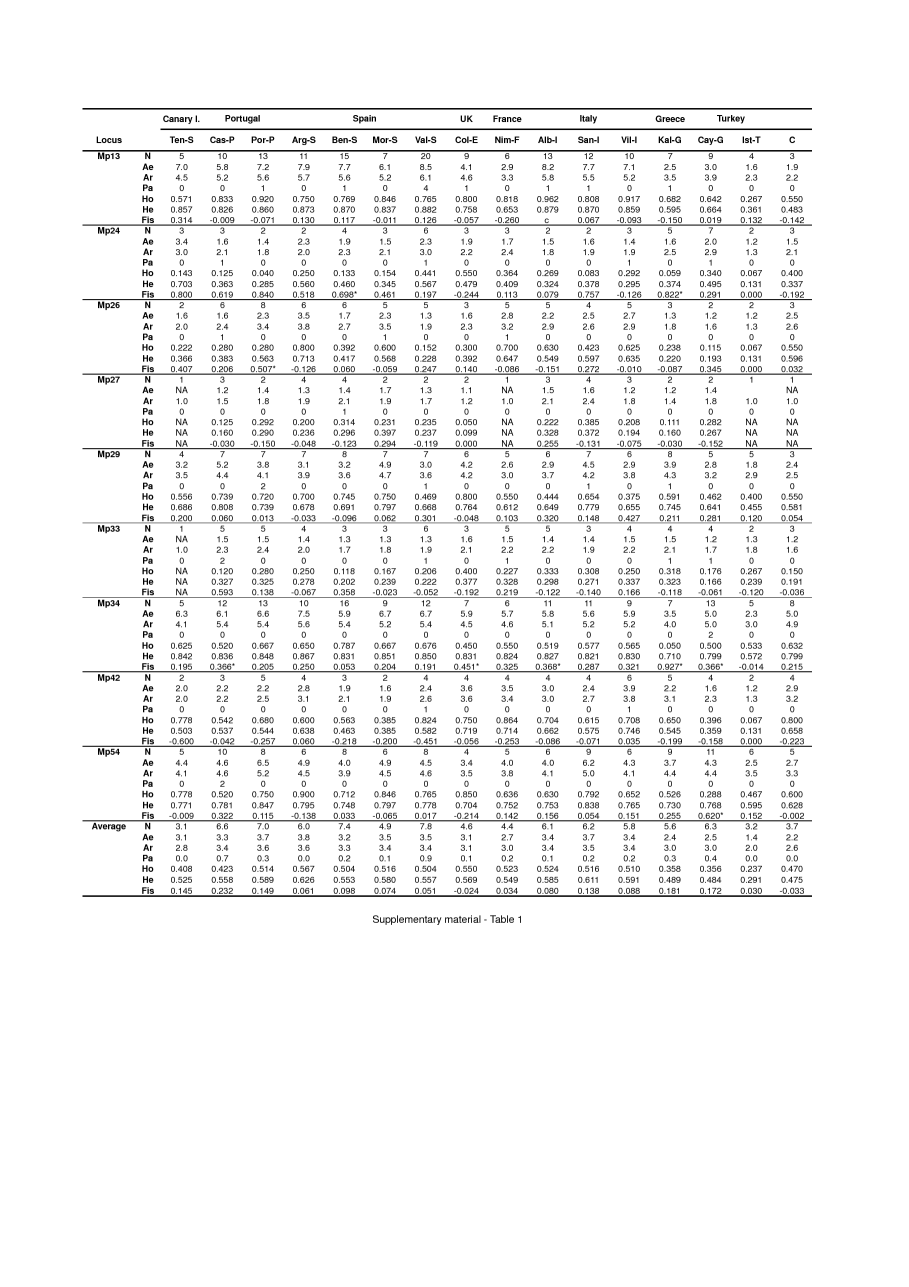

Supplement: Supplementary file 2 [file ece30002-3145-SD5.png]

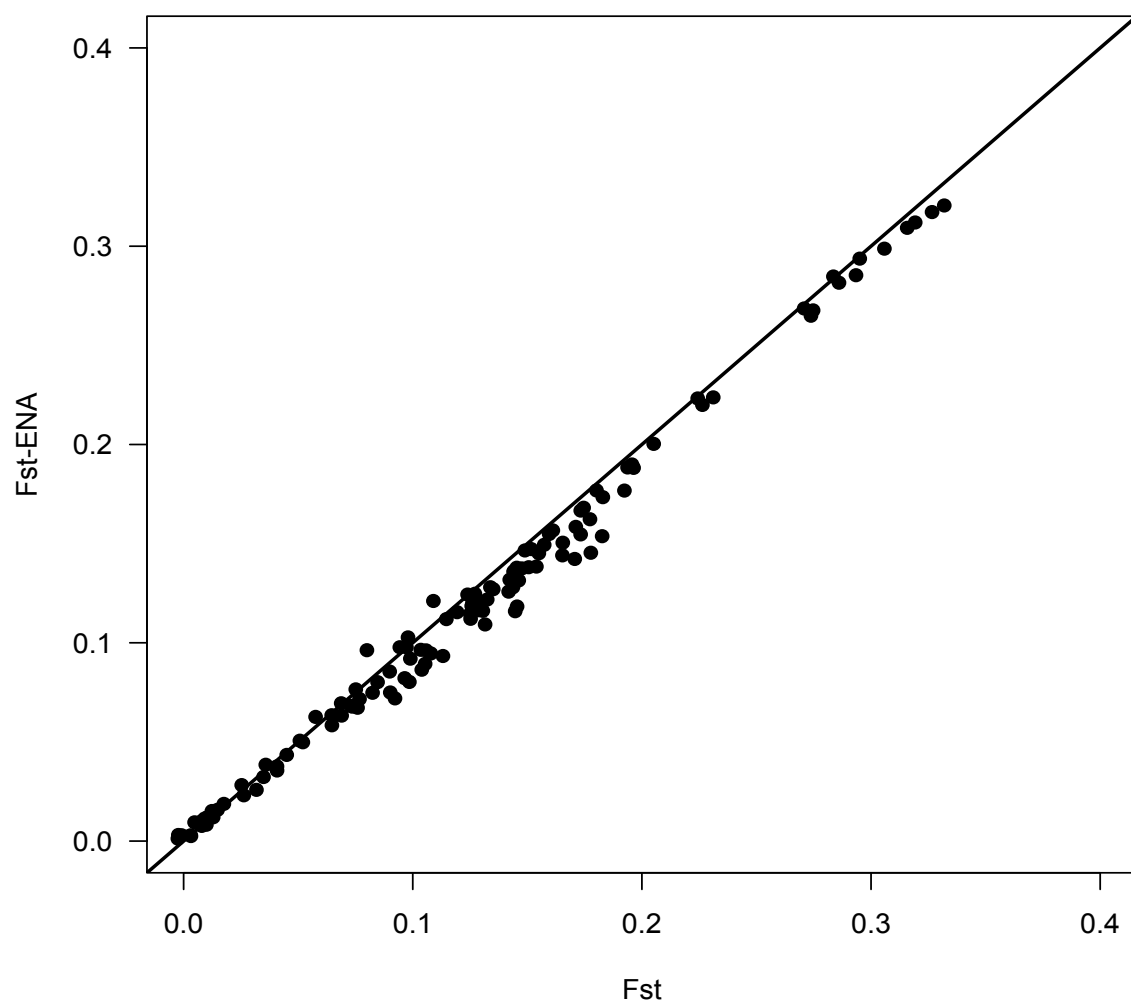

Supplement: Supplementary file 4 [file ece30002-3145-SD1.pdf]

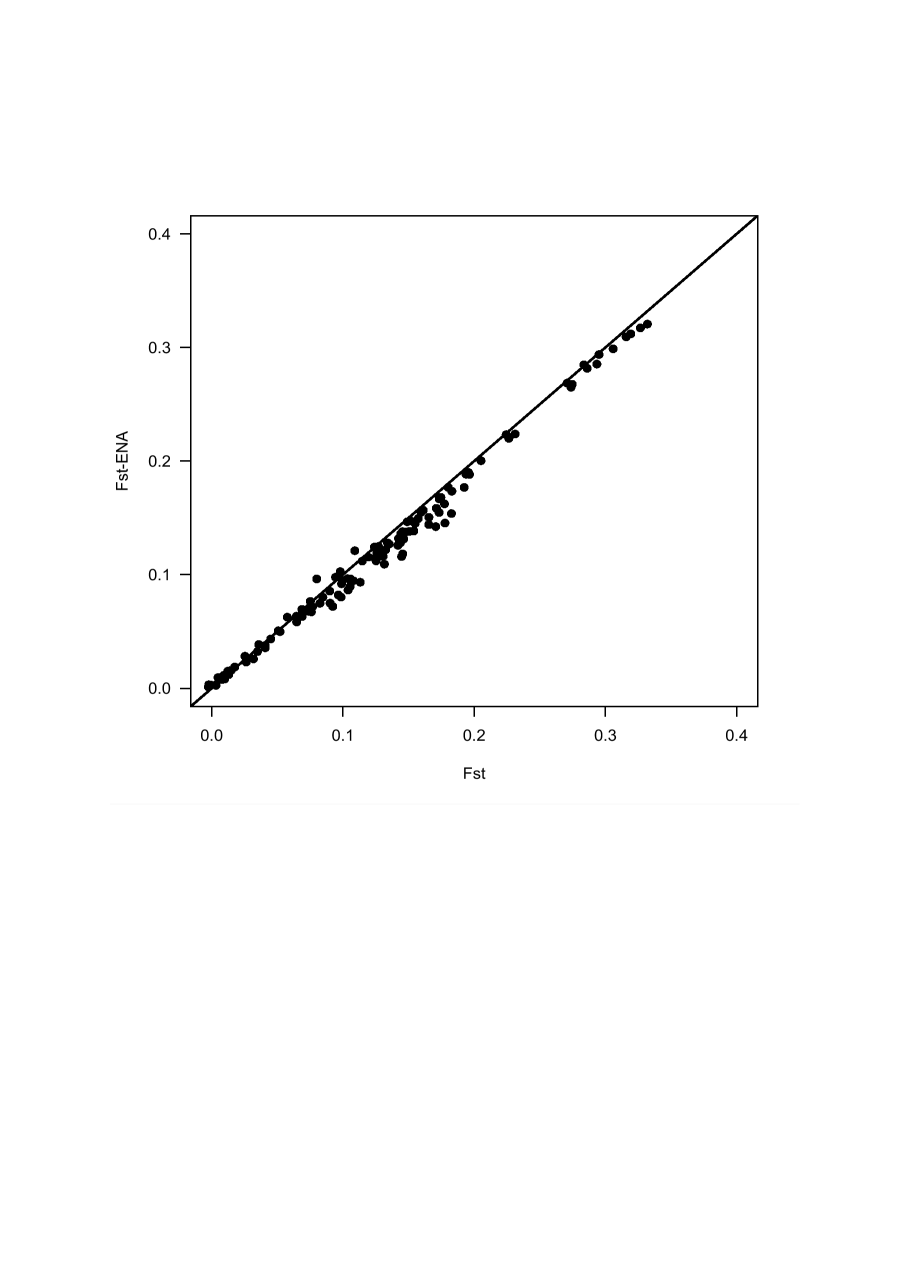

Supplement: Supplementary file 5 [file ece30002-3145-SD2.png]
